# Develop an emotion recognition system using jointly connectivity between electroencephalogram and electrocardiogram signals

**DOI:** 10.1016/j.heliyon.2025.e41767

**Published:** 2025-01-08

**Authors:** Javid Farhadi Sedehi, Nader Jafarnia Dabanloo, Keivan Maghooli, Ali Sheikhani

**Affiliations:** Department of Biomedical Engineering, Science and Research Branch, Islamic Azad University, Tehran, Iran

**Keywords:** Coupling EEG-ECG, Convolutional neural network (CNN), Effective connectivity, Emotion recognition, Transfer learning

## Abstract

This study pioneers an innovative approach to improve the accuracy and dependability of emotion recognition (ER) systems by integrating electroencephalogram (EEG) with electrocardiogram (ECG) data. We propose a novel method of estimating effective connectivity (EC) to capture the dynamic interplay between the heart and brain during emotions of happiness, disgust, fear, and sadness. Leveraging three EC estimation techniques (Granger causality (GC), partial directed coherence (PDC) and directed transfer function (DTF)), we feed the resulting EC representations as inputs into convolutional neural networks (CNNs), namely ResNet-18 and MobileNetV2, known for their swift and superior performance. To evaluate this approach, we have used EEG and ECG data from the public MAHNOB-HCI database through 5-fold cross-validation criterion. Remarkably, our approach achieves an average accuracy of 97.34 ± 1.19 and 96.53 ± 3.54 for DTF images within the alpha frequency band using ResNet-18 and MobileNetV2, respectively. Comparative analyses in comparison to cutting-edge research unequivocally prove the efficacy of augmenting ECG with EEG, showcasing substantial improvements in ER performance across the spectrum of emotions studied.

## 1 Introduction

Emotions are fundamental to human existence, influencing our cognition, decision-making, and interpersonal relationships. They deeply affect how we interpret our surroundings and play a vital role in maintaining both our psychological and bodily well-being. Recognizing and understanding emotions in others is crucial in fields such as psychology, marketing, and artificial intelligence (AI) [[1], [2], [3]]. Emotion recognition technologies have found applications in improving service of customers, developing smarter AI, and improving diagnosis of mental health. To measure emotional states, researchers use techniques like electrocardiography (ECG) [[4], [5], [6]] and electroencephalography (EEG) [[7], [8], [9], [10], [11]]. EEG monitors the electrical activity in the brain to detect patterns that correspond to different emotional states, while ECG assesses heart rate variability which can indicate relaxation levels or stress. These technologies facilitate a more profound insight into the biological foundations of emotions and aid in designing responses that are empathetically attuned to human sentiments.

There are some brain analysis tools such as connectivity that estimate information flow of lobes to distinguish mental states. This tool has three categories, structural, effective and functional [12]. If we consider anatomical structures and compute connectivity between different brain regions, it's structural category [12,13]. It is typically measured using techniques such as diffusion tensor imaging (DTI) which tracks the movement of water molecules along white matter tracts in the brain. This measure provides insights into the physical wiring of the brain, and offers information about the underlying architecture of brain networks. Also, it allows for the investigation of abnormalities in brain connectivity associated with neurological and psychiatric disorders. but it has disadvantages as limited in providing information about the dynamic functional interactions between brain regions, vulnerable to artifacts and limitations of imaging techniques. Also, it may not fully capture the complexity of brain function since structural connections do not always equate to functional connections. The second one, functional connectivity (FC) refers to the statistical correlation or synchronization of neural activity between different brain regions. It is typically measured using techniques such as functional magnetic resonance imaging (fMRI) or EEG [12,14]. In this technique, correlation does not imply causation, so functional connectivity measures may not reveal the directionality of interactions. Also, FC is susceptible to noise, artifacts, and confounding factors that can influence correlation measures and is limited in providing information about the underlying structural connections of the brain. The third technique, effective connectivity (EC) describes the influence one brain region exerts over another, often in a directed or causal manner [12,15]. It focuses on understanding the directional influence or causal relationships between brain regions. This technique provides insights into the directionality and causality of interactions between brain regions, enables the investigation of network dynamics and how information flows within the brain. Also, it is useful for modeling and understanding cognitive processes and brain disorders.

Convolutional Neural Networks (CNNs) have emerged as the most frequently used tool in EEG studies, primarily due to their robust ability to process and analyze spatial and temporal data [16,17]. This popularity is underpinned by CNNs’ exceptional proficiency in handling the complex and high-dimensional data typical of EEG recordings. Researchers favor CNNs because they effectively extract useful patterns and features from the raw EEG signals without requiring extensive handy crafted feature engineering. This capability is particularly advantageous in neuroscientific research, where accurate interpret of brain signals is crucial. CNNs are applied in a variety of EEG-related tasks such as classify specific pattern of wave, recognize classes of emotions [[7], [8], [9], [10], [11]], and diagnose neurological disorders [[18], [19], [20], [21]]. Their widespread adoption in the field is also facilitated by the availability of powerful computational tools and libraries that support CNN implementations, making them accessible to a broad spectrum of researchers and practitioners in neuroscience.

Generating 2D images for CNN applications frequently relates to transforming single-channel EEG into time-frequency (TF) representations. Commonly used single-channel techniques include the continuous wavelet transform (CWT) [7,18,[22], [23], [24], [25]], the Wigner-Ville distribution (WVD) [18], and the synchrosqueezing wavelet transform (SSWT) [10]. While these methods remain vital for signal analysis, multi-channel approaches, such as connectivity analysis of brain, have gained prominence for their broader applicability and richer insights into EEG data. These multi-channel methods provide a more integrated perspective on neural interactions in contrast to single-channel analyses.

TF representation methods, including CWT, WVD, and SSWT, specialize in examining the evolving frequency components of EEG and ECG signals over time. These approaches are particularly effective for single-channel data, enabling detailed exploration of temporal and frequency dynamics linked to diverse cognitive and physiological states. They offer a focused, granular understanding of neural activity.

On the other hand, brain connectivity analysis leverages data from multiple EEG channels to evaluate interactions across different brain regions. This macroscopic approach uncovers complex network dynamics and regional interplay during specific tasks. The comprehensive insights offered by connectivity analysis are particularly valuable for understanding how brain regions coordinate, an essential factor in diagnosing and treating neurological disorders.

In summary, while TF representation methods provide specific frequency analysis, brain connectivity approaches excel in delivering comprehensive insights into the networked activity of the brain, leading to more significant discoveries in EEG research.

### 1.1 Purpose of this study

The purpose of this study stems from the critical role emotions play in shaping human experiences, influencing cognitive processes, behaviors, and social dynamics. Accurately recognizing and understanding emotions is essential among different domains, including marketing, psychology and artificial intelligence. Emotion recognition technologies, particularly those utilizing EEG and ECG, offer profound insights into the physiological basis of emotions, enhancing applications in customer service, AI development, and mental health diagnostics. The study aims to advance these technologies by leveraging the capabilities of CNNs to process complex EEG data. CNNs are favored for their effectiveness in extracting meaningful patterns from high-dimensional data without extensive manual intervention. This research further explores the potential of multi-channel EEG approaches, such as brain connectivity analysis, over traditional single-channel time-frequency representation methods. By focusing on the interactions across multiple brain regions, this study seeks to provide a more comprehensive understanding of neural dynamics, ultimately contributing to improved diagnostic and therapeutic strategies for neurological disorders.

### 1.2 Contribution of this study

Contribution of this study is as follows:

- Estimate coupling of information flow between multi-channels of ECG and EEG signals while emotional processing task. In this regards, three EC method named partial directed coherence (PDC), Graner causality (GC) and direct transfer function (DTF) are extracted from 3 ECG channels and 32 EEG channels of four emotions (happy, fear, disgust and sad) from the public and well-known MAHNOB-HCI database. In our knowledge, this is the first time that ECG is added to EEG to estimate emotional connectivity from MAHNOB-HCI database.
- Extract deep features from incorporation of EEG-ECG windows and represent the novel interaction multichannel EC images (IMEC) in four mentioned emotional classes.

### 1.3 Related studies

Li et al. [26] computed the phase locking value (PLV), a functional connectivity (FC) metric, from 32 channels across DEAP and MAHNOB-HCI databases. They discriminate emotional classes— positive, neutral and negative —using the Graph Regularized Extreme Learning Machine and SVM techniques. Their approach achieved classification accuracies of 68 % for the MAHNOB-HCI dataset and 62 % for the DEAP dataset.

Moon et al. [27] introduced a CNN framework to capture neural activity patterns through a matrix of connectivity generated from three methods of transfer entropy (TE), phase locking value (PLV) and Pearson correlation coefficient (PCC). To enhance performance, the connectivity matrix was structured using two strategies to evaluate the resemblance and variation between EEG signals from various spatial regions. The system was investigated on EEG data from the DEAP dataset, achieving an accuracy value of 80.73 % with the PLV metric. The findings also revealed that the effectiveness of emotional video classification strongly depends on how accurately the connectivity matrix represents emotional valence, emphasizing that valence-specific connectivity improves classification outcomes.

Bagherzadeh et al. in Ref. [9] used two EC techniques of PDC and direct directed transfer function (dDTF) to discriminate five emotions. They achieved the average accuracy of 99.43 % and 96.26 % on MAHNOB-HCI dataset using ResNet-50 on dDTF and PDC images of alpha frequency band, respectively. This underscores the efficiency of the EC-based CNN model in detecting emotions from EEG signals. In Ref. [8], brain connectivity was assessed using PDC across five frequency bands to generate EC images. These images were then input into six different CNN models: ResNet-18, AlexNet, ShuffleNet, DarkNet-19, Xception and Inception-V3. The ResNet-18 model, achieved the best accuracy of 94.27 % on DEAP and 95.25 % for MAHNOB-HCI dataset. In Ref. [11] three EC methods of PDC, TE and dDTF are fused to created specific image to be used for combined pre-trained CNN models with long-short term memory (LSTM) models. Finally, they achieved the accuracy of 98.76 % and 98.86 % to classify emotions from the DEAP and MAHNOB-HCI datasets, respectively. Also, these methods are used to detect depression [28], schizophrenia [29] and mental workload [30] from EEG signals. For example, Mirjebreili et al. [28], generated EC images to feed hybrid pre-trained CNNs such as EfficientnetB0 and LSTM to predict response of MDD patients to treatment with selective serotonin reuptake inhibitors (SSRIs) antidepressants. Accuracy of this study was higher than 98 % on 30 Major Depressive Disorder (MDD) patients. This approach offers significant potential as a diagnostic tool for early MDD diagnosis and treatment planning. Bagherzadeh and Shalbaf in Ref. [29], estimated three EC measures of PDC, dDTF and TE and generate a fused image to feed pre-trained CNNs. They detect schizophrenia from EEG signals with the accuracy of higher than 95 %. Safari et al. in Ref. [30], estimated dDTF connectivity measure from EEG signals during low-task and high-task in mental workload. Then, selected best features by the minimum-redundancy-maximum-relevance (mRMR) technique and applied support vector machine (SVM) and achieved the accuracy of 89.53 %.

In our previous study [31], we have estimated GC from EEG-ECG with respect to eye-tracking data as gate. Then, we have generated images to feed to ResNet-18 and classify sadness and happiness from MAHNOB-HCI database [32]. The mean accuracy and area under the curve (AUC) achieved 91 % and 0.97, respectively. In this study, we consider more specific connectivity measures (PDC and DTF) along with GC without eye-tracking data. Also, we increase emotional classes from 2 to 4, sadness, happiness, fear and disgust.

## 2 Material and methods

### 2.1 MAHNOB-HCI dataset

This dataset represents a groundbreaking collection of EEG and ECG recordings obtained from 27 (16 female/11 male) various healthy participants. Data were captured over 32 channels as the participants viewed 20 distinct video clips [32]. The group included varying educations, ranging in age from 19 to 40 years (mean age = 26.06, SD = 4.39). Recordings adhered to local regulations and institutional protocols at Imperial College London, negating the need for ethical review and approval. EEG signals were collected using the advanced Biosemi Active II system, adhering to the internationally accepted 10–20 electrode placement standard, with a sampling frequency of 256 Hz. The electrodes were positioned at the following sites: Fp1, Af3, F3, F7, Fc6, Fc2, Cz, C4, Cp6, Cp2, Fc5, Fc1, P3, P7, P4, P8, Po3, O1, Oz, Pz, Fp2, C3, T7, Cp5, Cp1, Af4, Fz, F4, F8, T8, Po4, and O2. Additionally, three ECG leads were recorded concurrently from the upper right and left chest, and lower left abdomen, all sampled at the same frequency.

A distinctive three-phase protocol was devised to provoke specific emotional reactions. The procedure started with a neutral video to minimize any existing emotional conditions, continued with the display of emotionally stimulating clips, and ended with a self-report task. Each session spanned roughly 50 min, with personal evaluations lasting about two and a half minutes. The video segments varied in length, ranging from 34 to 117 s, ensuring participants remained attentive and experienced diverse emotional states. Participants assessed their emotions based on arousal and valence scales, as well as provided descriptive labels. For this research, samples were categorized by arousal and valence levels, ultimately centering on four emotions: happiness, disgust, fear, and sadness.

### 2.2 Proposed ER system using inter connectivity of EEG-ECG

**Fig. 1:**
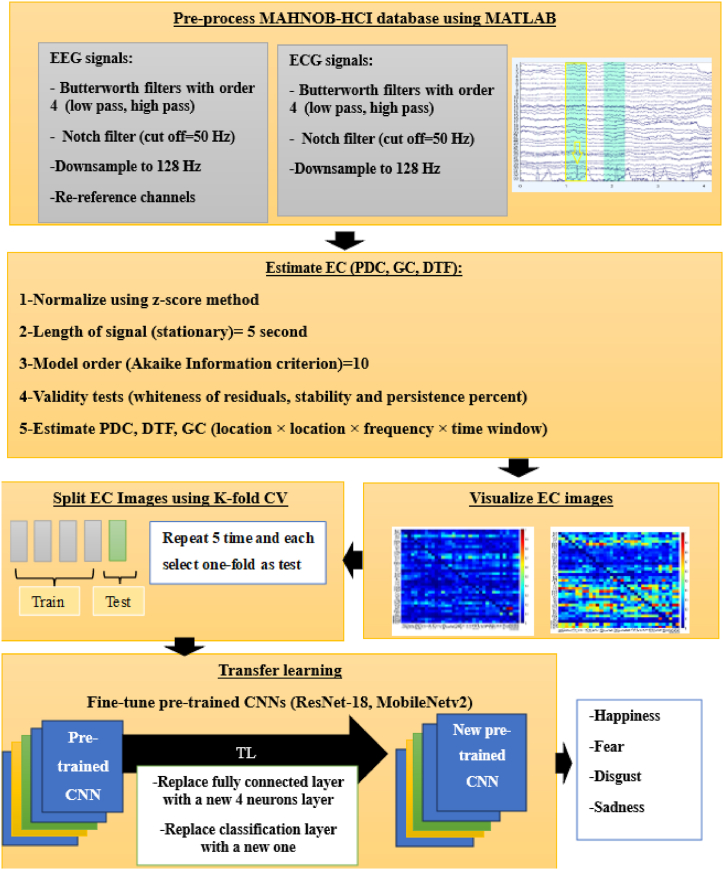
Flowchart of proposed IMEC-TL method to recognize four emotional classes.

In this study, the interplay between multiple ECG signals and EEG signals are considered in four basic emotional classes. These interactions are estimated through EC methods. Comparison of these methods show which one is more effective in recognition of these emotional classes. Flowchart of this study is shown in Fig. 1. First, both signals are filtered to remove noise and artifacts. Then, sampling frequency is reduced to lower computational burden and costs. After that, reference of EEG channels is changed by mean of all ones. After passing the preprocessing block, the EC measures are estimated. To estimate DTF, PDC and GC measures, stationarity of signals must be satisfied, therefore, signals are segmented into smaller parts. After that, each mentioned EC is estimated using a particular order of model and validated through defined test. Then, PDC, GC and DTF are visualized in color scales as images. Images are split into five-folds and two quick and high performance (MobileNetV2 and ResNet-18) pre-trained CNN models are re-trained. This approach is TL, as the knowledge from pre-trained CNN models is applied to our new task. This process involves replacing the fully connected layer with a new one containing 4 neurons, corresponding to the number of emotions in this study. Additionally, the classifier layer associated with the previous fully connected layer is substituted with a new one.

### 2.3 Estimate effective connectivity

EC serves as a valuable approach for examining multiple EEG signals to uncover the relationships between brain channels, enabling the differentiation of various brain states. Each EC technique evaluates these interactions through its unique perspective. GC, DTF and PDC are the most used EC methods in emotion recognition [8,9,11], detection of schizophrenia [33], object detection [34], detection of epilepsy [35] and vegetative state patients [36]. These three methods are employed here to uncover the interaction between three ECG channels and 32 EEG channels across four emotional states: fear, happiness, sadness, and disgust.

#### 2.3.1 Classical linear GC

During the simultaneous examination of two signals, denoted as x(t) and y(t), the second signal is considered to have a causal effect on the first if incorporating its historical data significantly enhances the prediction accuracy of the first signal compared to using only its own past information [36]. Within the framework of a univariate autoregressive (AR) model, this association is expressed by Eq (1), where $a_{i,j}$ denotes the coefficients of the model (commonly estimated via the least squares approach), P indicates the order of the autoregressive (AR) model, and $u_{i}$ represents the residuals. Notably, the forecast for each signal (x and y) depends solely on its own past values [37].

(1) $$\begin{array}{c} x\left(n\right)=\sum_{k=1}^{P}a_{x,k}x\left(n-k\right)+\sum_{k=1}^{P}u_{x}u\left(n\right)\\ y\left(n\right)=\sum_{k=1}^{P}a_{y,k}y\left(n-k\right)+\sum_{k=1}^{P}u_{y}u\left(n\right) \end{array}$$

The GC from y to x is calculate by Eq (2) as follow:

(2) $${GC}_{y\to x}=\ln\left(\frac{V_{x|\bar{x}}}{V_{x|\bar{x},\bar{y}}}\right)$$

The variances of these residuals are determined as $V_{x|\bar{x},\bar{y}}=var\left(\mu_{yx}\right)$ and $V_{x|\bar{x}}=var\left(\mu_{y}\right)$. The GC metric ranges from zero to infinity. As highlighted earlier, GC provides the benefit of asymmetry, making it suitable for detecting directional interactions. However, being a linear parametric approach, it relies on an autoregressive model with a specified order P.

For this research, GC was computed using MATLAB 2023b in combination with the HERMES connectivity toolbox, with the autoregressive model order set to 10.

#### 2.3.2 PDC

PDC is a method used to determine the directional flow of information between two EEG signals within the frequency domain. To estimate the PDC from the i th EEG channel to the j th channel at a specific frequency f, Eq (3) is employed, as referenced in literature [34,[38], [39], [40]]:

(3) $$\pi_{ij}\left(f\right)=\frac{{\bar{A}}_{ij}\left(f\right)}{\sqrt{\sum_{m=1}^{N}{\bar{A}}_{mj}\left(f\right){\bar{A}}_{mj}^{*}\left(f\right)}}$$

In this equation, $A_{ij}\left(f\right)$ represents the frequency-specific autoregressive coefficient from matrix A (denoted by $a_{ij}$), which is determined by Eq (4):

(4) $${\bar{A}}_{ij}\left(f\right)=\delta_{ij}-\sum_{r=1}^{P}a_{ij}\left(r\right)e^{-j2\pi fr}$$

Similarly, P stands for the order of the model, here we preset it to 10. The PDC is calculated using MATLAB (version 2023b) with the HERMES connectivity toolbox.

#### 2.3.3 DTF

DTF is estimated similarly as PDC [37]. Eq (5) estimate DTF. H is the transfer function matrix H (PDC uses those of Ᾱ). Its value is ranging from zero (no coupling between the two EEG signals) to one (complete coupling of the two EEG signals).

(5) $$DTF\left(f\right)=\frac{H_{ij}\left(f\right)}{\sqrt{h_{j}^{H}\left(f\right)h_{j}\left(f\right)}}$$

In a similar manner, DTF was calculated using MATLAB (version 2023b) via HERMES connectivity toolbox, with the P fixed at 10.

### 2.4 Transfer learning and pre-trained CNNs

Transfer learning is extensively applied in deep learning, leveraging pre-trained models as a starting point for addressing new challenges. Adapting a network through transfer learning is typically quicker and requires fewer resources compared to building a model from the ground up with randomly initialized parameters. This approach facilitates the effective utilization of pre-learned features for a novel task, even when dealing with a constrained set of training images [[8], [9], [10], [11],^41^].

**Fig. 2:**
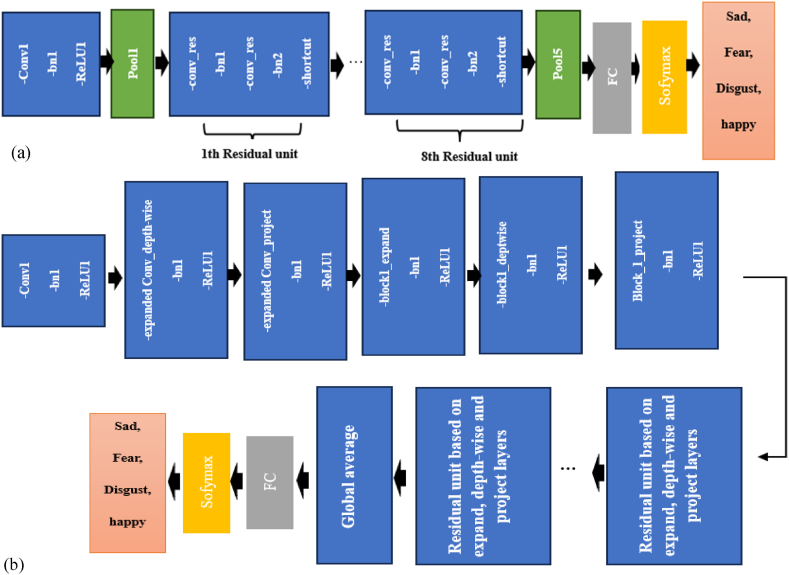
Simplified structure of (a) ResNet-18 and (b) MobileNetV2.

With the variety of transfer learning models available, it is crucial to assess trade-offs and ensure compatibility with the study's objectives. After thorough experimentation to determine the most appropriate models for our needs, MobileNet-v2 [42] and ResNet-18 [43] were chosen for their optimal balance between storage efficiency, computational demands, and practical applicability. ResNet-18 includes an initial 7 × 7 convolutional layers, followed by eight sequential residual blocks (each consisting of two convolutional layers with a filter size of 3 × 3 and a shortcut connection), and ends with a fully connected layer comprising 1000 neurons. Its structure is depicted in Fig. 2(a). MobileNet-v2 employs clipped rectified linear units (cReLU) with a value of 6 and builds upon residual blocks by incorporating depthwise convolutional layers. For detailed information about both CNN architectures, refer to Refs. [42,43]. Fig. 2 (b) illustrate simple structure of MobileNetV2.

### 2.5 Evaluation statistics

A 5-fold CV approach is employed to evaluate the classification performance of our IMEC-CNN method. For each EC category, the images are divud into 4 folds for re-training the two pre-trained CNNs and 1-fold for testing. This process is repeated five times, ensuring that every image is included in the testing phase exactly once. The mean accuracy and AUC are calculated as performance metrics using Eq (6) and Eq (7) for the 4-class emotion recognition task, with AUC computed in a binary format [44].

(6) $$average\;accuracy=\frac{\sum_{i=1}^{l}\frac{{tp}_{i}+{tn}_{i}}{{tp}_{i}+{tn}_{i}+{fp}_{i}+{fn}_{i}}}{l},i=1,2,3,4\text{.}$$

(7) $$AUC=\frac{1}{2}\left(\frac{TP}{TP+FN}+\frac{TN}{TN+FP}\right)$$

Where, in Eq (6), ${tp}_{i}$ and ${tn}_{i}$ the true positive and true negative for 4 classes (i=1,2,3,4). ${fp}_{i}$ and ${fn}_{i}$ are false positive and false negative for four emotional classes. In Eq (7), TP as the true positive, means correct decisions of class 1 against other three emotional classes, and TN as the true negative is true decisions of other three emotional classes against class 1. FN as false negative means wrong decision of three emotional classes against class 1 and FP as false positive means wrong decision of class 1 against other three emotional classes. This process is repeated for each four emotional classes to be as positive class.

## 3 Results

**Fig. 3:**
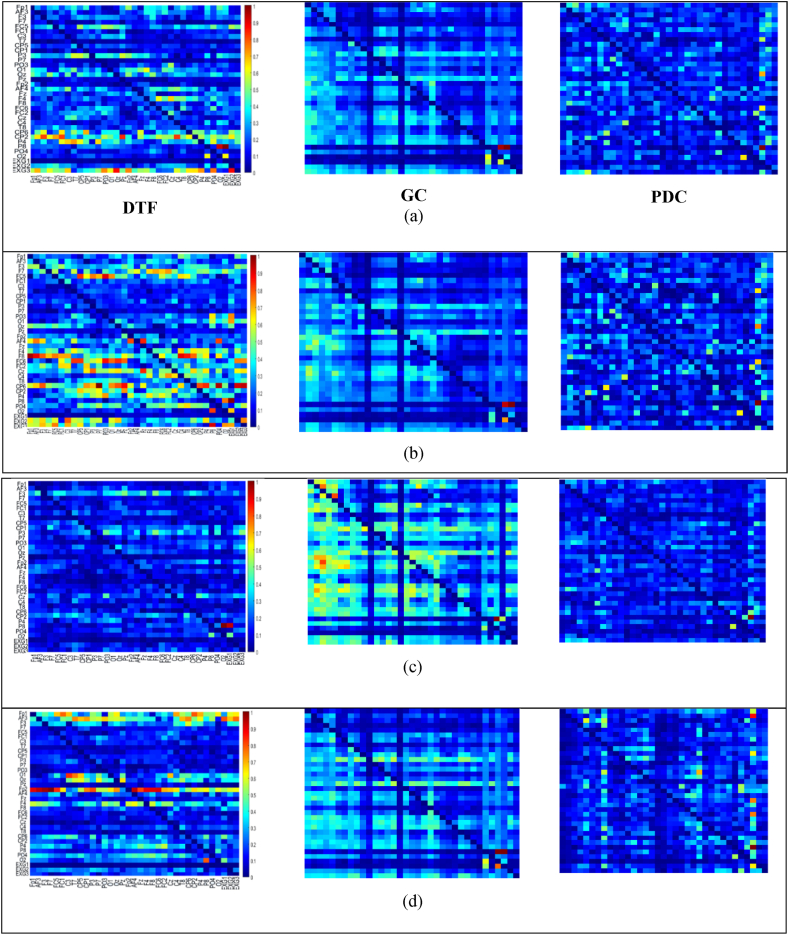
An example of DTF (left), GC (middle) and PDC (right) images for one subject at four emotions of (a) happiness, (b) disgust, (c) fear and (d) sadness. The color scales represent the information flow, where cooler blue shades correspond to lower values and warmer red hues indicate higher values. The initial 32 arrays along each axis correspond to EEG, while the final three represent ECG channels positioned at the upper right chest, upper left chest, and abdomen left side.

32 EEGs and 3 ECGs from the MAHNOB-HCI (27 participants) dataset underwent initial preprocessing via the EEGLAB toolbox within MATLAB software. Samples from three participants were removed due to technical mistakes. To deduce the GC, PDC and DTF measures among the cleaned EEGs from the MAHNOB-HCI (24 participants) database the procedure was employed. The window length was determined by the variance ratio test to ensure the selection of stationary segments. A consistent 5000 ms sliding window (with 50 % overlap) was chosen across all database to create a substantial number of images for models of pre-trained CNNs. The MVAR model order of 10, guided by the Akaike Information Criterion (AIC) [12], was uniformly used. The suitability of the MVAR was then verified through assessments of consistency, stability and residual whiteness measures [12]. Following these validations, GC, DTF and PDC EC measures were computed within five recognized frequency bands—theta, delta, beta, alpha, and gamma—using the 5000 ms windows. The resultant GC, DTF and PDC, represented as 35 × 35 matrices was used as an image for input into various pre-trained CNNs. Consequently, 2853 (sadness), 4391 (happiness), 2281 (fear) and 2750 (disgust), EC images were generated per frequency band from the MAHNOB-HCI database. Fig. 3 present DTF images (left), GC images (middle) and PDC images (right) from one participant across four emotions of happiness, disgust, fear and sadness in the best (alpha) frequency band from up to down, respectively. The color scales represent the transfer of data, where smaller values appear in cooler shades of blue, and larger values are shown in warmer hues of red. The first 32 arrays in each axis are for EEG (PO3, O1, C4,F7, FC5, FC1, Fp1, CP1, FC2, Pz, Fp2, AF4, Cz, T8, CP6, AF3, P3, P7, Oz, F8, CP5, FC6, F3, C3, Fz, F4, CP2, P4, P8, PO4, O2, T7) and the last three are for ECG (EXG1-3 are upper right and left corners of chest and left of abdomen, respectively) channels. Based on Fig. 3, DTF images illustrate the most interconnections between EEG and ECG than GC and PDC. Also, for this kind of image, these interactions are most in happiness (Fig. 3 (a)) and disgust (Fig. 3 (b)). Moreover, based on DTF images, there are high interactions between the third ECG signal and most EEG channels for happiness (right side of Fig. 3 (a)) and disgust (right side of Fig. 3 (b))) emotional class than fear (right side of Fig. 3 (c))) and sadness (right side of Fig. 3 (d)).

Subsequently, a comprehensive fine-tuning of two advanced pre-trained CNN models, SqueezeNet and ResNet-18 was undertaken using these images from the training set (4-folds) to identify four mentioned emotional classes, with test performed on 1-fold from the test set. Following this, average accuracy and AUC performance metrics were calculated for the test set images. This process was iterated for 5-times, thereby treating the images of each fold as unseen data to classify the four designated emotional classes. The overall performance was summarized using the average and standard deviation of the computed metrics. The training process utilized the cross-entropy loss function. Also, the adaptive moment estimation (ADAM) optimizer [45] was used. Key training hyperparameters featured a starting learning rate of 0.0008, a mini-batch size of 32, a gradient decay factor of 0.99 and a maximum of 30 epochs. All implementations and computations were performed using MATLAB 2023b on a system equipped with an Intel(R) Core(TM) i7-10610U processor (1.80 GHz) and an Nvidia Quadro P520 graphics card.

**Fig. 4:**
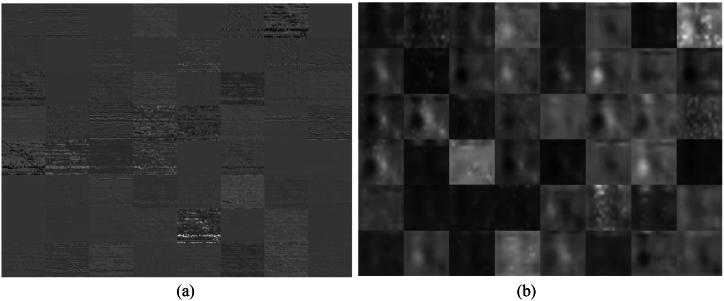
(a) 64 extracted deep feature maps from first convolutional layer (conv1) and (b) pool5 layer of ResNet-18 on a DTF images from sadness for one subject. This image illustrate that each filter is not appropriate.

As mentioned in section 2.4, the first convolutional layer of ResNet-18 extracts 64 feature maps for an input image. Activations of this layer for the happy-alpha-DTF image is shown in Fig. 4 (a). As it can be observed, all of these feature maps are not appropriate. Fig. 4 (b) shows activation of the specific last layers name ‘pool5’ for that image.

**Fig. 5:**
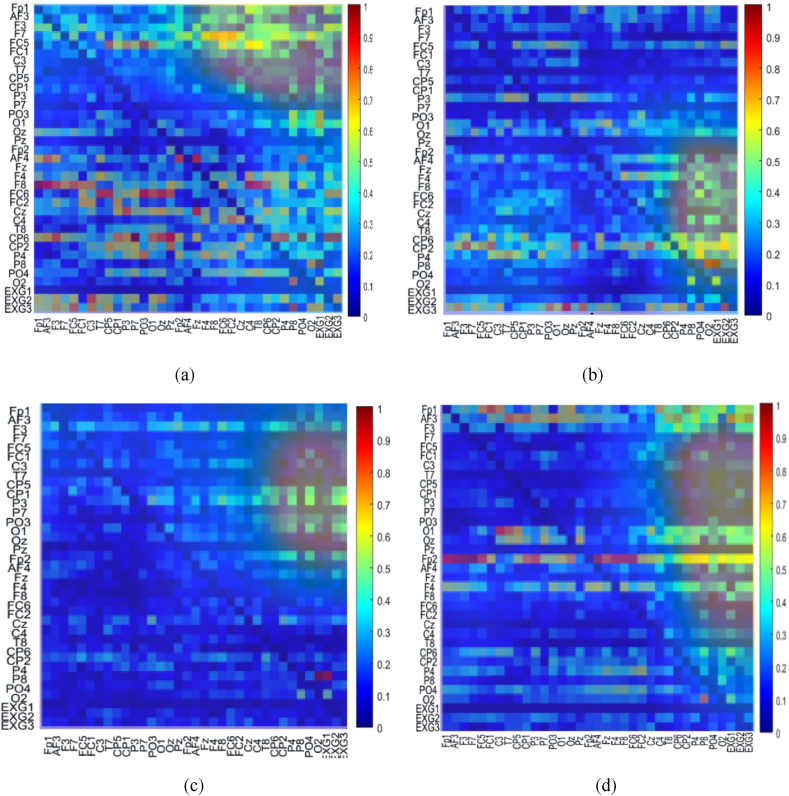
An example from Grad-Cam activation functions maps of ResNet-18 for alpha (best) frequency band on alpha-DTF images (a) disgust, (b) happiness, (c) fear and (d) sadness for a subject.

Fig. 5 shows the results of the gradient-weighted class activation mapping (Grad-CAM) method applied to alpha-DTF images for the sadness class (left side of Fig. 3). Grad-CAM generates a gradient-weighted class activation map that shows how changes in the classification score of input X affect the network's evaluation of a specific class [46]. This tool is useful for elucidating how a network makes its predictions, ensuring it concentrates on the relevant segments of the data. This technique leverages the gradients of the network's classification score relative to its last convolutional feature map. Regions in the data that have high values on the Grad-CAM map are critical in influencing the network's score for a particular class. Therefore, Fig. 5 shows how ResNet-18 decides which belongs to each emotional class. Also, this figure reveals that interaction of EEG and ECG channels are effective on the final decision about recognition of emotions. For example, for disgust class, the ResNet-18 observes upper right side of image, for happy observes lower right side of DTF, for fear observes middle right side and for sad observes bigger parts from right side, from up to down right side. According to Fig. 5 (a) the second and third ECG channels (EXG2 and EXG3) are coupled with all EEG channels. Also, most of EEG channels are coupled with EXG3 according to Fig. 5 (b). According to Fig. 5 (c), there is low coupling between ECG and EEG channels in fear. Two channels from the frontal, i.e., Fp2 and Fp1, are connected with all EEG and ECG channels during sadness (Fig. 5 (d)).

**Fig. 6:**
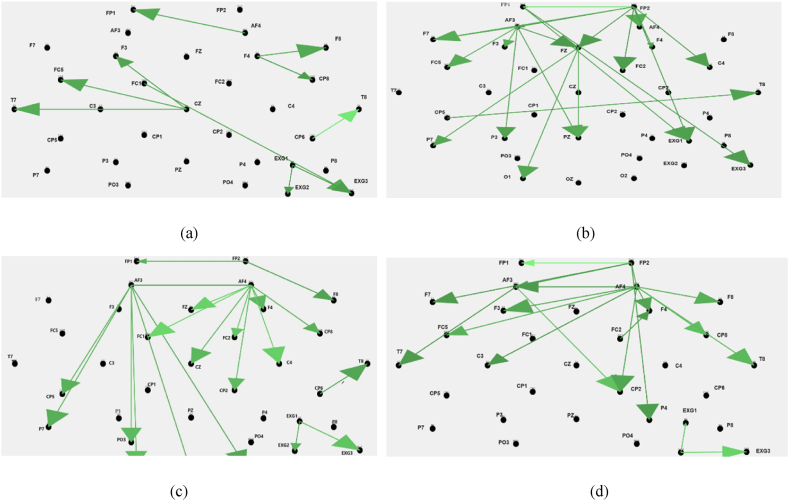
Topographic representation of DTF at alpha frequency band for disgust (a), happiness (b), fear (c) and sadness (d) for 3rd subject at one-five second segment.

Fig. 6 shows topographic images of DTF at alpha frequency band for disgust (a), happiness (b), fear (c) and sadness (d) for 3rd participant. Duration of signal in this image is 5 s. This pattern of interconnection through EEG and ECG was alike for majority of participants. As evident from Fig. 6(a) and (b), there are coupling between Fp1, Fp2 and Fc1 EEG channels and EXG3 channel (ECG). Fig. 6 (c) and 6 (d) reveals no interaction between ECG and EEG channels in this moment.

Table 1, Table 2 report accuracies obtained using the ResNET-18 and MobileNetV2 on all kinds of IMEC images per frequency bands, respectively. Based on Table 1, the highest accuracy obtained via alpha-DTF and ResNet-18 equal to 97.34 with standard deviation of 1.19. After that, beta and gamma frequency bands for DTF images using this CNN achieved high accuracies of 95.94 % and 95,26 %, respectively.

**Table 1:** Average accuracy (mean ± std) of ResNet-18 on IMEC images from five frequency bands.

**Image**	**BAND**	**Accuracy (%)(mean ± std)**
PDC	Delta	84.65 ± 3.45
	Theta	86.35 ± 3.62
	Alpha	88.54 ± 2.07
	Beta	86.76 ± 2.80
	gamma	86.45 ± 2.65
GC	Delta	85.53 ± 3.87
	Theta	86.08 ± 3.65
	Alpha	89.54 ± 2.12
	Beta	87.56 ± 3.56
	gamma	87.28 ± 2.76
DTF	Delta	88.87 ± 3.63
	Theta	90.69 ± 2.98
	Alpha	97.34 ± 1.19
	Beta	95.94 ± 1.57
	gamma	95.26 ± 2.86

**Table 2:** Average accuracy of MobileNetV2 on IMEC images from five frequency bands.

**Image**	**BAND**	**Accuracy (%)(mean ± std)**
PDC	Delta	82.89 ± 3.01
	Theta	86.12 ± 2.28
	Alpha	87.29 ± 3.01
	Beta	84.92 ± 2.30
	gamma	83.47 ± 3.25
GC	Delta	84.22 ± 3.20
	Theta	85.38 ± 2.80
	Alpha	89.09 ± 3.24
	Beta	88.13 ± 2.22
	gamma	86.63 ± 2.60
DTF	Delta	86.72 ± 3.63
	Theta	89.28 ± 3.09
	Alpha	**96.53** ± **3.54**
	Beta	94.78 ± 2.03
	gamma	93.83 ± 3.26

**Fig. 7:**
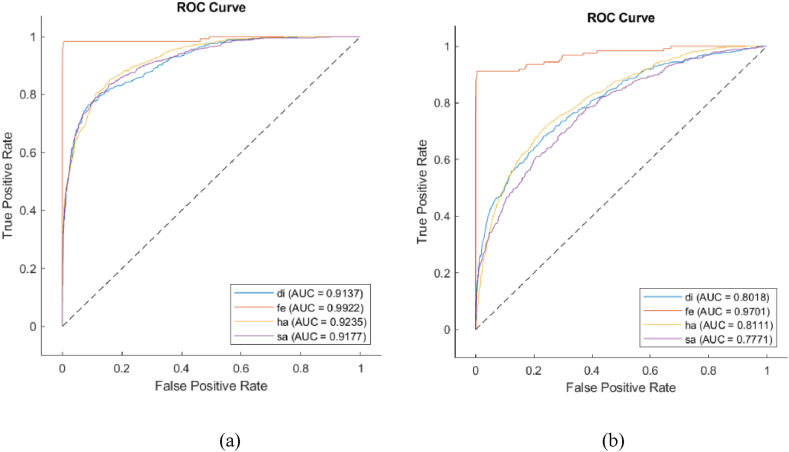
ROC curves for DTF images of alpha frequency band using (a) ResNet-18 (winner model) and (b) MobileNetv2 (runner model).

Based on Table 2, the highest accuracy value obtained using alpha-DTF images and MobileNetV2 equal to 96.53 % (std = ± 3.54). After that, beta and gamma frequency bands for DTF images using this CNN achieved high accuracies of 94.78 % and 93.83 %, respectively. Fig. 7 (a) and (b) shows ROC for best result, alpha-DTF-ResNet-18 and the second-best model, MobileNetV2, respectively. Based on Fig. 7 (a), AUC for happiness, sadness, fear and disgust for this setup are 0.92, 0.91, 0.99 and 0.91. According to Fig. 7 (b), MobileNetV2 achieved the AUC values of 0.97, 0.81, 0.80 and 0.77 for fear, happy, disgust and sad, respectively.

**Fig. 8:**
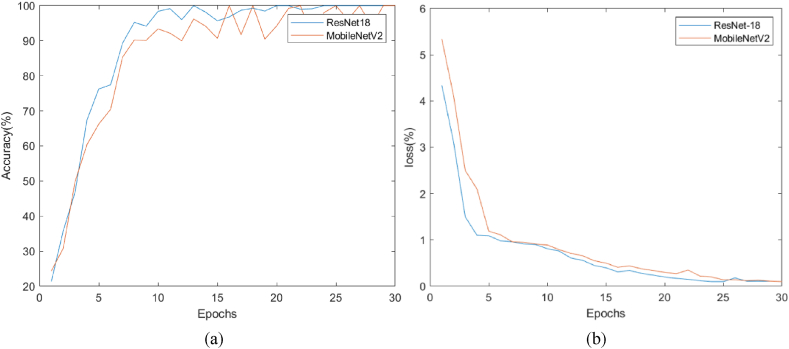
(a) Accuracy and (b) loss curves for 30 epochs for ResNet-18 (blue color) and MobileNetV2 (brown color) on training set of alpha-DTF images.

Fig. 8 (a) and 8 (b) illustrates progress of training based on accuracy (percent) and loss function across epochs, respectively. This figure shows the result of training ResNet-18 on DTF from the alpha (optimal) frequency band. Observing this figure demonstrate superiority of ResNet-18 against MobileNetV2 based on accuracy and loss values.

## 4 Discussion

The aim of this study is developing an ER system based on interaction of multichannel EEG and ECG signals in point of connectivity measures. To our knowledge, this is the first time that connectivity measures are estimated jointly from EEG and ECG for ER domain. EEG and photoplethysmogram (PPG) has been used to calculate both gPDC and dDTF during deception in interview [47,48].

The coupling of ECG and EEG measurements in emotion processing tasks provides a more comprehensive perception of the physiological basis of emotions. Latest study highlights the potential of combining these two modalities to achieve a richer, multidimensional insight into emotional states. EEG, which measures brain electrical activity, and ECG, which records the heart's electrical signals, are both influenced by emotional stimuli. EEG captures the brain's direct response to emotional inputs through various frequency bands like alpha, beta, and gamma, which have been linked to different emotional processes. In contrast, ECG provides data on the autonomic nervous system's response to emotions. Moreover, integrating ECG and EEG is beneficial for instantaneous emotion classification frameworks, such as those used in mental health monitoring and neuromarketing. These systems benefit from the rapid response detection of EEG and the stability and simplicity of ECG measurements, making them more robust and user-friendly in everyday applications. The continuous advancements in sensor technology and data analysis techniques are likely to further enhance the efficacy and practicality of EEG-ECG combined studies in emotion research, offering deeper undertesting about the intricate relationship between the brain and the heart in emotions.

Comparing three kinds of EC images based on Table 1, Table 2, DTF outperforms the other two. DTF's advantage lies in its comprehensive frequency-domain analysis, ability to normalize interactions, and distinguishing direct versus indirect influences, making it particularly valuable in detailed network connectivity analyses in neuroscience and other fields that handle complex signal systems.

Our analysis revealed that the alpha frequency wave stands out as the most significant among various frequency bands, based on Table 1, Table 2 This frequency band yielded the highest accuracy in recognizing four specific emotional classes from MAHNOB-HCI database, using highly optimized CNNs. Other frequency bands, such as beta, gamma, theta, and delta, followed in subsequent ranks. These results align with findings from other research [8,15,49,50], which indicate that the alpha, beta, and gamma bands are closely linked to emotional processes. Furthermore, multiple psychological studies have emphasized the alpha band's key role in emotional processing, noting its association with relaxed states [[51], [52], [53], [54]].

According to Fig. 5, ECG channels are coupled with most frontal EEG channels, some parietal (P8, Po4), central (Cp5, Cp1,C3) and temporal (T7). Frontal region has been introduced as the origin of emotional processing area in physiological studies. Also, these channels are similar with introduced channels in Refs. [7,9,10].

When comparing ResNet-18 and MobileNetV2 across all IMEC images, ResNet-18 outperforms MobileNetV2 in terms of performance. Although both CNNs utilize a residual unit design, MobileNetV2 incorporates a rectified linear unit (ReLU) clipped at 6. This modification results in fewer parameters, making it faster and more suitable for mobile applications. Additionally, MobileNetV2 employs depthwise convolution. Despite these advantages, as evidenced by the data in Table 1, Table 2 and Fig. 7, ResNet-18 demonstrates superior accuracy and AUC values across all frequency bands.

### 4.1 Comparison with recent researches on MAHNOB-HCI

Table 3 compares our results with recent ER researches on same dataset (MAHNOB-HCI). The discrepancies between this study and them lie in emotional classes, modalities and evaluation criteria. We have integrated the ECG modality into our ER framework to explore the interconnection between the brain and the heart through various emotions. Additionally, we propose a multimodal framework for recognizing emotional states by simultaneously processing both EEG and ECG modalities. We used the GC connectivity measure to analyze the information flow between brain regions and the heart during basic emotions.

**Table 3:** Comparison of ER studies on MAHNOB-HCI database.

**Ref**	**Modality**	**Method**	**Number of classes**	**Evaluation criterion**	**Accuracy (%) (m ± std)**
[8]	EEG	Alpha-PDC	5	LOSO^a^	95.25 ± 2.23
[9]	EEG	Alpha-dDTF, ResNet-50	5	10-fold CV	96.26 ± 0.53
[11]	EEG	Fusion of TE, PDC, dDTF, CNN-LSTM	5	LOSO	98.86 ± 0.57
[55]	EEG	MSST, deep CNN	4	Hold out	88.17
[56]	ECG	1DCNN-LSTM	2^b^	Hold out	73.33 (valence), 60 (arousal)
[57]	EEG	Entropies, Sugeno-fuzzy inference system	4	Hold out	85.20 %
**Ours**	EEG-ECG	IMEC, **ResNet-18**, MobileNetV2	4	5-fold CV	97.34 ± 1.19

a LOSO = leave-one-subject-out.

b valence (high valence/low valence), and arousal (high arousal/low arousal).

Unlike other strategies that generate time-frequency images of multivariate synchrosqueezing transform (MSST) from a single channel [55] or use raw single channel data [56], our approach is multichannel. This multichannel method provides a more comprehensive representation of EEG signals, which is then used to train pre-trained CNNs.

According to Table 3, our results demonstrate significant improvement. When compared to Ref. [56], which used ECG signals to classify valence and arousal classes, our accuracy is notably higher (97.34 % vs. 73.33 % for valence and 60 % for arousal). Similarly, when compared to Refs. [8,9,55,57], which used EEG signals, our method also outperforms them (97.34 % vs. 95.25 %, 96.26 %, 85.2 % and 88.17 %, respectively).

Based on Table 3, our results surpass all of these studies except for Bagherzadeh et al. [11], who achieved higher accuracy by generating brain connectivity images using a fusion of three methods (TE, PDC, and dDTF) and combining CNN and LSTM. Our use of multichannel and multimodal approaches has led to higher accuracy in our ER framework compared to recent studies.

### 4.2 Advantage, disadvantage and limitation of this study

Advantages of this study are as follows:

• Estimate coupling of ECG and EEG WHILE emotions as a way to improve ER systems performance.
• Jointly represent EC of multichannel ECG and EEG to investigate their involvement in sadness, happiness, disgust and fear.
• Use transfer learning approach to recognize emotions from physiological data due to insufficient number of samples for train from the first.

Here, a restriction is that it processes entire segments pertaining to emotional clips, which may not always be relevant. This is because there may be instances where the subject's attention diverges from the clip, leading to distraction, or situations where multiple emotions are elicited simultaneously.

The second restriction is that, examination of variability of heart rate is a common approach in several ER researches. Since HRV directly change during emotions such as horror, anger and disgust. Therefore, this could be a useful metric in our study, but its frequency range is below 4 Hz, and the corresponding EEG range is delta. Delta is not the specific frequency band in emotion classification procedure. Hence, HRV is not considered in this study. The other limitation is number of samples, the database has 27 subject and each one has watched 18 emotional video clips.

## 5 Conclusion and upcoming work

This study pivots on enhancing the accuracy of emotion classification frameworks by integrating EEG data with ECG data to calculate EC, capturing the brain-heart interaction during emotions such as happiness, fear, sadness and disgust. To achieve this, three EC estimation techniques—GC, DTF, and PDC—were applied, and their outputs were used as inputs for advanced pre-trained CNNs, specifically ResNet-18 and MobileNetV2. The highest average accuracy recorded was 97.34 ± 1.19 % with DTF images in the alpha frequency band using ResNet-18, and 96.53 ± 3.54 % with MobileNetV2. These findings highlight that combining EEG with ECG data significantly improves emotion recognition performance compared to existing methods, particularly for emotions like happiness, disgust, fear, and sadness.

In the future, we would use the eye track data as a gate to choose most effective parts of EEG and ECG data during sadness, happiness, disgust and fear. Also, we would combine other deep learning techniques like LSTM to add time information to our proposed ER system.

## CRediT authorship contribution statement

**Javid Farhadi Sedehi:** Writing – review & editing, Writing – original draft, Visualization, Validation, Software, Methodology, Investigation, Formal analysis, Conceptualization. **Nader Jafarnia Dabanloo:** Writing – review & editing, Validation, Supervision, Methodology, Investigation, Conceptualization. **Keivan Maghooli:** Writing – review & editing, Validation, Supervision, Methodology, Investigation, Conceptualization. **Ali Sheikhani:** Validation, Methodology, Conceptualization.

## Data availability

In this study, the public available MAHNOB-HCI database was analyzed. The databases can be found here: https://mahnob-db.eu/hci-tagging/.

## Ethics declaration

In this study, the public available MAHNOB-HCI database was analyzed. The study involving human participants was conducted in compliance with local legislation and institutional guidelines for of Imperial College London, thereby exempting it from ethical review and approval.

## Declaration of competing interest

The authors declare that they have no known competing financial interests or personal relationships that could have appeared to influence the work reported in this paper.
